# Coupled and Synchronization Models of Rhythmic Arm Movement in Planar Plane

**DOI:** 10.3390/bioengineering9080385

**Published:** 2022-08-12

**Authors:** Affiani Machmudah, Denys Dutykh, Setyamartana Parman

**Affiliations:** 1 Faculty of Advanced Technology and Multidiscipline, Kampus C Jalan Mulyorejo, Universitas Airlangga, Surabaya 60115, Indonesia; 2 Research Center for Hydrodynamics Technology, National Research and Innovation Agency (BRIN), Jl. Hidro Dinamika, Keputih, Sukolilo, Surabaya 60112, Indonesia; 3 Univ. Grenoble Alpes, Univ. Savoie Mont Blanc, CNRS, LAMA, 73000 Chambéry, France; 4 Fakulti Teknologi Kejuruteraan Mekanikal dan Pembuatan, Universiti Teknikal Malaysia Melaka, Durian Tunggal, Melaka 76100, Malaysia

**Keywords:** nonlinear dynamics, chaotic behavior, coupled system, synchronization, biomechanics modeling, rhythmic movement, healthy movement system

## Abstract

Nonlinear dynamics have become a new perspective on model human movement variability; however, it is still a debate whether chaotic behavior is indeed possible to present during a rhythmic movement. This paper reports on the nonlinear dynamical behavior of coupled and synchronization models of a planar rhythmic arm movement. Two coupling schemes between a planar arm and an extended Duffing-Van der Pol (DVP) oscillator are investigated. Chaos tools, namely phase space, Poincare section, Lyapunov Exponent (LE), and heuristic approach are applied to observe the dynamical behavior of orbit solutions. For the synchronization, an orientation angle is modeled as a single well DVP oscillator implementing a Proportional Derivative (PD)-scheme. The extended DVP oscillator is used as a drive system, while the orientation angle of the planar arm is a response system. The results show that the coupled system exhibits very rich dynamical behavior where a variety of solutions from periodic, quasi-periodic, to chaotic orbits exist. An advanced coupling scheme is necessary to yield the route to chaos. By modeling the orientation angle as the single well DVP oscillator, which can synchronize with other dynamical systems, the synchronization can be achieved through the PD-scheme approach.

## 1. Introduction

Bodily rhythm is an essential part of human life [1]. In terms of physical movement, everyday performances, such as walking, running, swimming, dancing, sports, and other life activities, frequently employ the repetition of motion. When a person loses the ability to conduct a rhythmic performance, it is not only a sign of an unhealthy phase of the human body system, but it will also significantly disturb the quality of the person’s life. For example, in the case of post-stroke injury, the person most likely will lose the ability to perform complex body movements that involve repetitive motion. 

The history of movement variability in biomechanics research can be traced to Bernstein’s report in 1967. When humans perform two identical movements, the trajectories of the first movement are never repeated in the second movement [2]. This simple phenomenon is evidence of the variability in human movement, which Bernstein used the term “repetition without repetition” to express it. Since this Bernstein report, the study of the variability in human movement, which is commonly referred to as Bernstein’s problem [2,3,4], has become a principal research interest in the field of human movement science, biomechanics, and human gait. Movement variability reflects that there are many possible solutions to achieve an identical movement. Then, how the human brain chooses the solution to achieve the desired motion among those possible solutions is one of the very challenging questions [5]. The chosen trajectories are potentially highly affected by the health conditions of a human musculoskeletal system. Thus, understanding movement variability is necessary to obtain a healthy movement system. It is also part of the biomechanics of sports to achieve the best performance of athletes in sports activities. Furthermore, there are many health movement issues related to repetitive movements that remain questionable. Among them are the post-stroke injuries that create difficulties when moving a patient, and Parkinson’s disease, which generates oscillating hand motions. 

Traditionally, movement variability is modeled as system error or noise so that it is considered an undesirable condition corresponding to health issues [6,7]. In line with the development of the nonlinear tools of dynamical system theory, recent research has modeled movement variability using the nonlinear dynamics approach, which considers the variability in human movement to be the essential factor for a healthy movement system. Using the dynamical system perspective, movement variability is unavoidable and inherent to the system [6]. The chaos tools typically used in deterministic chaos can be applied to investigate the chaotic behavior of human movement variability [8]. 

Experimental investigations have shown that humans tend to perform chaotic behavior during hand movements [9,10]. The chaotic behavior of human variability has been explained by the Lipsitz and Goldberger hypothesis [11], stating that a healthy system is characterized by its adaptability and flexibility to everyday stresses on human body parts. Aging has been seen as a factor in the loss of the complexity or capability to conduct chaotic behavior. More recently, Stergiou et al. [8,12,13] proposed a hypothesis stating that the healthy state of the human movement system was characterized by optimal variability in the form of a chaotic structure. 

Despite the certainty that nonlinear dynamics have become a very auspicious approach for better understanding the nonlinear behavior of the human movement system; there is still a debate whether movement variability presents due to the chaotic behavior of the human movement system or the chaotic pattern is detected due to the noise of the data measurement [6,14,15]. This is due to the fact that the ODE system of human motion control is typically not explicitly known [15,16], and nonlinear tools are employed on the data obtained from experimentally-based measurements. Furthermore, the results of LE, which is the most common nonlinear tool used to measure the chaotic structure of movement variability, are heavily influenced by the length of the data [15]. Thus, a study of the nonlinear dynamics of human rhythmic movements using the ODE system is necessary. The ODE system specifies the deterministic rule under which a variety of dynamical behavior can be clearly observed.

The aim of the present study is to investigate the modeling approach by which it is possible that the chaotic behavior appears during repetitive planar arm movements. This is necessary to understand the nonlinear phenomenon of human rhythmic movements to achieve a healthy movement system. To the best of the authors’ knowledge, this is the first time that the ODE system involving a hand posture model is used to study the rhythmic movements of the human arm.

Nonlinear oscillators have been used to interpret biological systems, which are commonly related directly or indirectly to physiological rhythms, such as circadian clocks [17], physiological stress [18], and bipolar disorder [19]. The coupled system and synchronization are very common phenomena in biological systems, which typically exhibit rhythmic behaviors. The coupled system reflects the connections between two or more entities that depend on each other. Coupling strength can be represented mathematically by a coupling constant. In the field of movement science, the coupled system has been used to model jellyfish locomotion [20], human gaits [21], animal gaits [22], and interlimb coordination [23]. 

When compared with previous approaches, where a nonlinear oscillator is directly used to predict the joint trajectories of rhythmic motion without considering the ODE system of the kinematics posture, this paper employs the kinematic differential ODE system that represents the gait and locomotion of the 3-link planar arm. Because the biological system can interact with the environment or other natural systems by making a coupling, the first model is the coupled system of the human arm system with the nonlinear dynamical system, which may represent the nonlinear phenomenon during hand movement activities. The ODE system established from the robotic approach, i.e., 3-DOF planar series manipulator motion, was employed to model the planar arm system. Since the ODE system of the human arm system has been established, it can be coupled with the dynamical system, which is likely present during the movement. The extended DVP oscillator was applied to represent such a dynamical system in coupling with the planar arm system. This paper selects the extended DVP oscillator because it has diverse applications in the simulation and modeling of nonlinear phenomena [24,25,26]. As suggested by Lipsitz and Goldberg [11], where the capability to handle uncertainty conditions is very important for a healthy life, the extended DVP system employed in this paper represents the uncertainty during repetitive movements.

Movement variability is observed based on the interval analysis of the angle domain. In this paper, the repetitive movement under consideration is in the form of the repetitive motion of the end-effector planar arm. It should be noted that the term repetition in Bernstein’s problem can be in different forms. For example, Beek et al. [23] consider rhythmic movement to be the interlimb coordination between two hands. 

In the biological system of living organisms, one cell system can synchronize with other cell systems or even with the environment and create a complex biological system. For example, a small zone in the right atrium of the heart is composed of thousands of pacemaker cells, which are connected to each other in order to maintain normal cardiac rhythm [1]. The second model is the synchronization of the planar human arm system with the extended DVP system, implementing the PD-scheme approach. Synchronization is a fundamental phenomenon in engineering and biological systems [1]. Since the work of Pecora and Carroll [27], the synchronization of the chaotic system has become a critical research topic because of its many possible applications in engineering, physics, and science [28]. They observed that between two identical chaotic systems, there was a possibility of synchronizing with each other when they shared information in the correct way. Recently, the control-system-based approaches have joined the research into the synchronization of the dynamical system [25,26,27,28,29]. In this paper, to study synchronization in the rhythmic arm movement, the orientation angle is modeled as the single-well DVP oscillator, and the PD-scheme synchronization approach is employed. 

The rest of the paper is organized as follows: Section 2 presents the mathematical modeling of the planar arm system. The coupled system model of the rhythmic arm movement is described in Section 3, and there are two coupling schemes developed. Section 4 presents the synchronization of the planar arm system with the extended DVP oscillator using the PD scheme. Section 5 presents the results and discussions. The parameter *k*, which exhibits chaotic behavior, is observed using the LE, the Poincare section, the phase space, and the heuristic approach. The response system of the synchronization model is investigated. The conclusions are presented in Section 6. 

## 2. System Model

Previous research has reported that the redundant planar series manipulator can be used to model the human arm [30,31]. Lee and Bang [30] modeled the human arm as a 3-link planar series manipulator to design the optical mouse, which can eliminate the coordinate disturbances that occur during skilled strokes. Ghosal [31] confirmed that the human arm can be modeled as a redundant serial manipulator and that the redundancy can be obtained from the Jacobian matrix null-space. For the serial manipulator, the redundancy is essentially kinematic [32]. Thus, this paper employs a 3-link planar open kinematic chain.

### 2.1. Mathematical Modeling to Observe Chaotic Behavior of Repetitive Arm Movement

**Figure 1:**
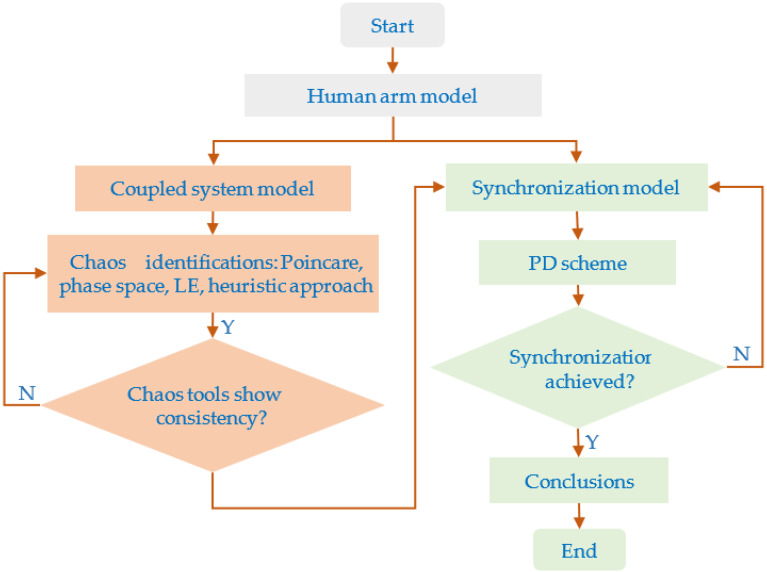
Computational method to observe the chaotic behavior of the rhythmic arm movement.

This work is an attempt to understand the phenomenon of chaotic behaviors that are present in human arm movements based on nonlinear dynamics and chaos theory. The ODE system was established from the 3-link planar series manipulator for the planar arm system, while the uncertainty was obtained from the nonlinear chaotic system. Figure 1 illustrates the mathematical modeling approach to achieve this goal. Firstly, the coupled system model was developed, and the chaos tools, which are the Poincare map, the phase space, the LE, and the heuristic approach, were employed to observe the dynamical behavior of the coupled system. To confirm the chaotic behavior, these chaos tools should show consistent results. After the nonlinear dynamics of the rhythmic arm motion had been obtained, the synchronization of the rhythmic arm movement was studied by employing the PD scheme. 

Nowadays, the study of variability in human movement is well-known as Bernstein’s problem [2,3,4]. This paper expresses the repetition term in Bernstein’s problem as the repetition of the end-effector path of the planar arm system. The rhythmic movement of the end-effector path in the Cartesian coordinate can be expressed as follows:

(1) $$x_{e}\left(t\right)=f\left(\varphi \right)\cdot \varphi \left(t\right);\;y_{e}\left(t\right)=g\left(\varphi \right)\cdot \varphi \left(t\right)$$

where $x_{e}\left(t\right)$, $y_{e}\left(t\right)$, φ, *t*, f(φ), and g(φ) are the desired end-effector positions on the *x*-axis, the desired end-effector position on the *y*-axis, angle of curve, time, parametric function f(φ), and parametric function g(φ), respectively. 

The motion was performed at a constant angular frequency *Ω*, as in the following:

(2) φ=Ωt

### 2.2. 2nd ODE of Kinematics Planar Arm Model

The second ODE of this planar arm system can be expressed as follows:

(3) $$\ddot{\theta }=J^{-1}\left(\ddot{\chi }-\dot{J}\dot{\theta }\right)$$

(4) $$\theta ={\left[\theta_{1}\;\;\theta_{2}\;\;\theta_{3}\;\;\right]}^{T};\;\theta_{g}=\theta_{1}+\theta_{2}+\theta_{3};\;\chi ={\left[x\;\;y\;\;\theta_{g}\;\;\right]}^{T}$$

(5) $$J=\left[\begin{array}{ccc} -l_{1}sin\theta_{1}-l_{2}sin\left(\theta_{1}+\theta_{2}\right)-l_{3}sin\theta_{g} & -l_{2}sin\left(\theta_{1}+\theta_{2}\right)-l_{3}sin\theta_{g} & -l_{3}sin\theta_{g}\\ l_{1}cos\theta_{1}+l_{2}cos\left(\theta_{1}+\theta_{2}\right)+l_{3}cos\theta_{g} & l_{2}cos\left(\theta_{1}+\theta_{2}\right)+l_{3}cos\theta_{g} & l_{3}cos\theta_{g}\\ 1 & 1 & 1 \end{array}\right]$$

(6) $$j=\left[\begin{array}{ccc} -l_{1}{\dot{\theta }}_{1}cos\theta_{1}-l_{2}cos\left({\dot{\theta }}_{1}+{\dot{\theta }}_{2}\right)\left(\theta_{1}+\theta_{2}\right)-l_{3}{\dot{\theta }}_{g}cos\theta_{g} & -l_{2}\left({\dot{\theta }}_{1}+{\dot{\theta }}_{2}\right)cos\left(\theta_{1}+\theta_{2}\right)-l_{3}{\dot{\theta }}_{g}cos\theta_{g} & -l_{3}{\dot{\theta }}_{g}cos\theta_{g}\\ -l_{1}sin\theta_{1}-l_{2}sin\left({\dot{\theta }}_{1}+{\dot{\theta }}_{2}\right)\left(\theta_{1}+\theta_{2}\right)-l_{3}{\dot{\theta }}_{g}sin\theta_{g} & -l_{2}sin\left({\dot{\theta }}_{1}+{\dot{\theta }}_{2}\right)\left(\theta_{1}+\theta_{2}\right)-l_{3}{\dot{\theta }}_{g}sin\theta_{g} & -l_{3}{\dot{\theta }}_{g}sin\theta_{g}\\ 0 & 0 & 0 \end{array}\right]$$

where $\theta_{i}$, $\theta_{g}$, *x*, *y*, *t*, $l_{i}$_,_ *J*, ${\dot{\theta }}_{i}$, ${\ddot{\theta }}_{i}$ $\dot{J}$, $J^{-1}$, χ, and $\ddot{\chi }$ are the joint angle of the *i*th link, the orientation angle, time, *i*th link length, the Jacobian of forward kinematics, the first derivative of $\theta_{i}$, the second derivative of $\theta_{i}$, the first derivative of *J*, the matrix inverse of *J*, the state variables of inverse kinematics, and the second derivative of χ, respectively. 

Constraints for end-effector repetitive motion:

(7) $$\left(x,y\right)=\left(x_{e},y_{e}\right)\;x=l_{1}cos\left(\theta_{1}\right)+l_{2}cos\left(\theta_{1}+\theta_{2}\right)+l_{3}cos\left(\theta_{1}+\theta_{2}+\theta_{3}\right)\;y=l_{1}sin\left(\theta_{1}\right)+l_{2}sin\left(\theta_{1}+\theta_{2}\right)+l_{3}sin\left(\theta_{1}+\theta_{2}+\theta_{3}\right)\;x_{e}=f\left(t\right);\;y_{e}=g\left(t\right)$$

Constraints of joint angle limits:

(8) $$\theta_{imin}<\theta_{i}<\theta_{imax}$$

where *x, y*, $\theta_{imin}$ and $\theta_{imax}$ are the actual end-effector position on the *x*-axis, actual end-effector position on the *y*-axis, and the minimum and maximum joint angles of the *i*th link, respectively. 

The end-effector path was fixed during the repetitive movement, i.e., (*x, y*) = (*x_e_, y_e_*), while the orientation angle of the ODE system, ${\ddot{\theta }}_{g}$, needs to be defined. 

### 2.3. Inverse Kinematics (IK) Solution

Using a geometrical approach, the IK solution of the 3-link planar open kinematic chain can be obtained in the following [33]:

(9) $$c_{2}=A_{p}cos\left(\theta_{g}-\phi_{p}\right)+k_{p}$$

(10) $$A_{x}=\frac{-l_{3}x}{l_{1}l_{2}};\;A_{y}=\frac{-l_{3}y}{l_{1}l_{2}};\;A_{p}=\sqrt{A_{x}{}^{2}+A_{y}{}^{2}};$$

$$\phi_{p}=atan2\left(A_{y},A_{x}\right);\;k_{p}=\frac{r^{2}+l_{3}{}^{2}-l_{2}{}^{2}-l_{1}{}^{2}}{2l_{1}l_{2}};\;r=\sqrt{x^{2}+y^{2}}$$

(11) $$s_{2}=\pm \sqrt{1-c_{2}{}^{2}}$$

(12) $$\theta_{2}=atan2\left(s_{2},c_{2}\right)$$

(13) $$\theta_{1}=atan2\left(s_{1},c_{1}\right)$$

where $c_{2}$, $s_{2}$, $c_{1}$, $s_{1}$, $\phi_{p}$, $k_{p}$, $A_{p}$, and *r* are the sinus of $\theta_{2}$, cosine of $\theta_{1}$, sinus of $\theta_{1}$, cosine of $\theta_{2}$, phase shift, vertical shift, amplitude, and radius from the fix base, respectively. 

**Figure 2:**
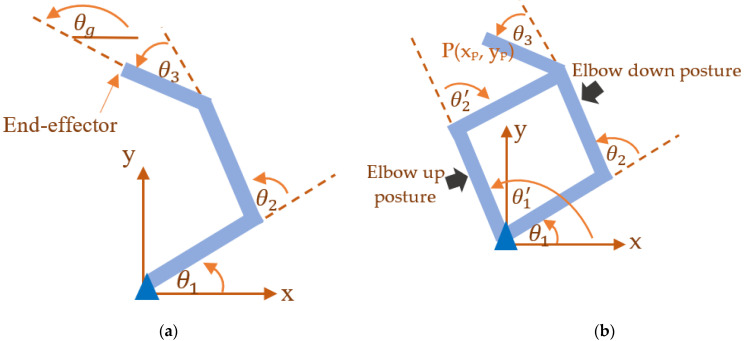
(**a**) Three-link planar system. (**b**) Elbow up and elbow down postures from the IK solution.

Figure 2a illustrates the planar arm system. There are three joint angles of the planar arm system: $\theta =\left[\begin{array}{ccc} \theta_{1} & \theta_{2} & \theta_{3} \end{array}\right]$. Figure 2b shows two possible postures related to elbow up and elbow down positions obtained from the inverse kinematics geometrical solution. 

The details of the IK equations are provided in Appendix A. It should be noted that there are many possible postures for the end-effector position *P(x_p_, y_p_)* in the workspace area of the 3-link planar arm system. Using a geometrical approach, the chosen trajectories were represented by the orientation angle, $\theta_{g}$. The corresponding joint angles of the first, second, and third links could be obtained using the IK solutions. For each chosen orientation angle, it corresponded to two possible postures, which were the elbow-up and elbow-down positions, as obtained from the IK solution.

### 2.4. Second Joint Angle Velocity

The analytic velocity of $\theta_{2}$ or the first derivative of $\theta_{2}$ can be expressed as follows:

(14) $${\dot{\theta }}_{2}=\frac{\partial \theta_{2}}{\partial x}\frac{dx}{dt}+\frac{\partial \theta_{2}}{\partial y}\frac{dy}{dt}+\frac{\partial \theta_{2}}{\partial \theta_{g}}\frac{d\theta_{g}}{dt}$$

From Equation (12), the derivative of $\theta_{2}$ can also be expressed as:

(15) $${\dot{\theta }}_{2}=\frac{\partial \theta_{2}}{\partial c_{2}}\frac{dc_{2}}{dt}$$

where:

(16) $$\frac{\partial \theta_{2}}{\partial c_{2}}=-\frac{1}{\sqrt{1-c_{2}^{2}}}$$

Partial derivatives for the second joint angle $\theta_{2}$_,_ which were derived using an algebraic method, are as follows:

(17) $$\frac{\partial \theta_{2}}{\partial \chi }=-\frac{1}{\sqrt{1-c_{2}^{2}}}\frac{\partial c_{2}}{\partial \chi }$$

The details of components $\frac{\partial c_{2}}{\partial \chi }$ are in the following:

(18) $$\frac{\partial \theta_{2}}{\partial x}=\frac{\partial \theta_{2}}{\partial c_{2}}\frac{\partial c_{2}}{\partial x}=\frac{-1}{\sqrt{1-c_{2}^{2}}}\frac{\partial c_{2}}{\partial x}\;\frac{\partial \theta_{2}}{\partial y}=\frac{\partial \theta_{2}}{\partial c_{2}}\frac{\partial c_{2}}{\partial y}=\frac{-1}{\sqrt{1-c_{2}^{2}}}\frac{\partial c_{2}}{\partial y}\;\frac{\partial \theta_{2}}{\partial \theta_{g}}=\frac{\partial \theta_{2}}{\partial c_{2}}\frac{\partial c_{2}}{\partial \theta_{g}}=\frac{-1}{\sqrt{1-c_{2}^{2}}}\frac{\partial c_{2}}{\partial \theta_{g}}$$

where:

(19) $$\frac{\partial c_{2}}{\partial x}=\left(\frac{l_{3}}{l_{1}l_{2}}\frac{xcos(\theta_{g}-\phi }{\sqrt{x^{2}+y^{2}}}-\frac{l_{3}}{l_{1}l_{2}}\frac{ysin(\theta_{g}-\phi }{\sqrt{x^{2}+y^{2}}}+\frac{x}{2l_{1}l_{2}\sqrt{x^{2}+y^{2}}}\right)\;\frac{\partial c_{2}}{\partial y}=\left(\frac{l_{3}}{l_{1}l_{2}}\frac{ycos(\theta_{g}-\phi }{\sqrt{x^{2}+y^{2}}}-\frac{l_{3}}{l_{1}l_{2}}\frac{xsin(\theta_{g}-\phi }{\sqrt{x^{2}+y^{2}}}+\frac{y}{2l_{1}l_{2}\sqrt{x^{2}+y^{2}}}\right)\;\frac{\partial c_{2}}{\partial \theta_{g}}=Asin(\theta_{g}-\phi )$$

The details of the derivations of ${\dot{\theta }}_{2}$ are in Appendix B. 

### 2.5. Domain of the Orientation Angle

Without considering the joint limit, the domain of $\theta_{g}$ could be determined by solving the equation in Equations (11) and (17) as follows:

(20) $$\left|c_{2}\left(\theta_{g}\right)=A_{p}cos\left(\theta_{g}-\varphi_{p}\right)+k_{p}\right|<1$$

The above equation shows that the solutions of the second ODE of this planar arm system exist in the boundary of the orientation angle, $\theta_{g}$. 

Since the joint angle of the planar arm has the joint limit, Equation (8), the $\theta_{g}$ boundary covers the solutions of Equation (20), which intersect the domain of the joint angles as follows:

(21) $$\theta_{i}\cap D_{\theta_{i}}$$

where $D_{\theta_{i}}$ is the domain or the operational area of the *i*th joint angle. 

### 2.6. General Solutions of ODE

Since $\left(x_{e},\;y_{e}\right)$ is a fixed path, $c_{2}$ is a bounded function. There is the $\theta_{g}$ boundary, and any arbitrary function, $\theta_{g}\left(t\right):R\to R$, generated inside the boundary of $\theta_{g}$ are possible solutions:

(22) $$\theta_{g}\left(t\right)\;\;\;\partial \theta_{g}$$

where $\theta_{g}\left(t\right)$ is an arbitrary function of time and $\partial \theta_{g}$ is the boundary of $\theta_{g}$.

The $\theta_{g}$ boundary considering the joint limits should be computed during the rhythmic motion. The computation can be performed iteratively for all of the end-effector trajectories in such a way so that Equation (21) is achieved.

## 3. Coupled Systems

Two coupling schemes are presented in this section to observe the dynamical behavior of the rhythmic movement of the planar human arm system. The conceptual model of the developed coupled system is adopted from the Coupled Human-Environment System (CHES). The CHES models the inseparable interaction between human systems and environment systems. This concept is also well-known as the Coupled Human and Natural System (CHANS) [34]. Both human systems and environment/natural systems are connected through certain schemes. How the processes of human systems and natural systems create an interaction, i.e., how they are coupled, is the research interest to understand such complex real phenomena.

**Figure 3:**
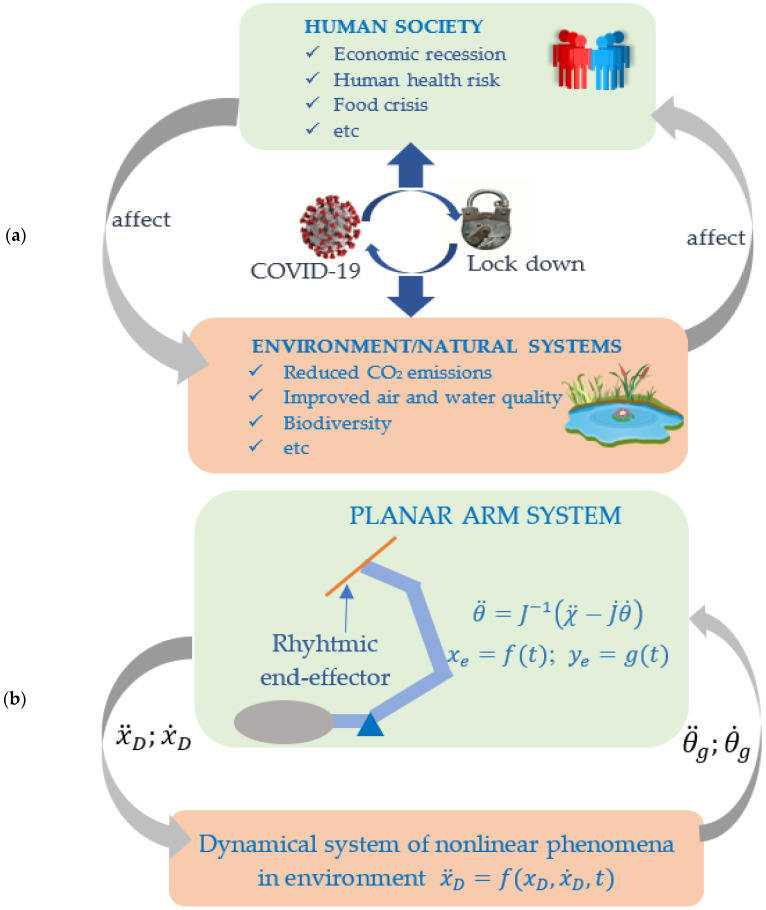
(**a**) Conceptual model of CHES of amid COVID-19 [35] (**b**) Coupled system model of rhythmic arm movement adopted from CHES/CHANS concept.

Figure 3a illustrates the CHES amid the COVID-19 crisis, as proposed by Sarkar et al. [35]. Figure 3b shows illustrations of the coupled system between the human arm planar system and the dynamical system of the nonlinear phenomenon in the environment using a bidirectional coupling scheme. As illustrated in Figure 1, the chaos tools were used to observe how chaotic behavior makes it possible to present a solution to human locomotion during repetitive hand movements. This paper focuses on the research questions of the possibility that chaotic behavior appears in repetitive hand movements. The end-effector of the planar arm is moved following the periodic path. The coupled system model that can yield the chaotic solutions of joint angle trajectories is investigated. 

To represent the nonlinear phenomenon in the environment, the extended DVP oscillator with two periodic forces [24] was employed:

(23) $${\ddot{x}}_{D}=\mu \left(1-x_{D}^{2}\right){\dot{x}}_{D}-\omega_{0}^{2}x_{D}-\alpha x_{D}^{3}-\gamma x_{D}^{5}+F_{1}cos\omega_{1}t+F_{2}cos\omega_{2}t$$

where *μ*, $\omega_{0}$, *α*, *γ, F_i_*, $\omega_{i}$ are real parameters. 

From the IK solution, it has been clearly shown that movement variability appears in the form of the orientation angle, $\theta_{g}$, so this variable should be further explored in the modeling approach. Thus, for the planar arm system, the coupling scheme was investigated via the orientation angle components, which can be in the form ${\dot{\theta }}_{g}$ and/or ${\ddot{\theta }}_{g}$.

### 3.1. Scheme 1

The first coupling scheme was studied when the planar arm system shared the information of the velocity to the nonlinear oscillator as follows:

(24) $${\dot{x}}_{D}=\frac{\Sigma \theta_{i}}{d}=\frac{Χ\left(4\right)+Χ\left(5\right)+Χ\left(6\right)}{d}$$

(25) $${\ddot{\theta }}_{g}=d\left(\mu \left(1-x_{D}^{2}\right){\dot{x}}_{D}-\omega_{0}^{2}x_{D}-\alpha x_{D}^{3}-\gamma x_{D}^{5}+F_{1}cos\omega_{1}t+F_{2}cos\omega_{2}\right)$$

where *d* is the coupling parameter. 

The state variable was defined as follows:

(26) $$Χ={\left[\begin{array}{cccccccc} \theta_{1} & \theta_{2} & \theta_{3} & {\dot{\theta }}_{1} & {\dot{\theta }}_{2} & {\dot{\theta }}_{3} & u & v \end{array}\right]}^{T}$$

With the above state, the first ODE form of the coupled system is in the following:

(27) $$\dot{Χ}=\left[\begin{array}{c} {\left[\begin{array}{ccc} Χ\left(4\right) & Χ\left(5\right) & Χ\left(6\right) \end{array}\right]}^{T}\\ J^{-1}\left(\ddot{\chi }-\dot{J}\dot{\theta }\right)\\ v\\ \mu \left(1-u^{2}\right)v-\omega_{0}^{2}u-\alpha u^{3}-\gamma u^{5}+F_{1}cos\omega_{1}t+F_{2}cos\omega_{2} \end{array}\right]$$

where:

$$\chi ={\left[\begin{array}{ccc} x & y & \theta_{g} \end{array}\right]}^{T};\;\;\;u=x_{D};\;\;v={\dot{x}}_{D}$$

$${\ddot{\theta }}_{g}=d\left(\mu \left(1-u^{2}\right)v-\omega_{0}^{2}u-\alpha u^{3}-\gamma u^{5}+F_{1}cos\omega_{1}t+F_{2}cos\omega_{2}\right)$$

### 3.2. Scheme 2

A further modification of scheme-1 was applied in scheme-2 by adding the nonlinear term to the DVP system and the coupling parameter to the orientation angle acceleration. 

Adding the nonlinear term in the DVP system was obtained as follows:

(28) $${\ddot{x}}_{D}=\mu \left(1-u^{2}\right)v-\omega_{0}^{2}u-\alpha u^{3}-\gamma u^{5}+F_{1}cos\omega_{1}t+F_{2}cos\omega_{2}+0.1\left({\dot{\theta }}_{g}sin\theta_{g}\right)$$

Orientation angle acceleration is augmented by the nonlinear coupling scheme with coupling constant *k* as follows:

(29) $${\ddot{\theta }}_{g}=k\left\{2\left(\mu \left(1-u^{2}\right)v-\omega_{0}^{2}u-\alpha u^{3}-\gamma u^{5}+F_{1}cos\omega_{1}t+F_{2}cos\omega_{2}\right)+0.1\left({\dot{\theta }}_{g}sin\theta_{g}\right)\right\}$$

where *k* is the coupling constant.

Equation (24) is still applied so that there are two parameters, which are *d* and *k*. 

Using scheme-2, the first ODE form can be expressed as follows:

(30) $$\dot{Χ}=\left[\begin{array}{c} {\left[\begin{array}{ccc} Χ\left(4\right) & Χ\left(5\right) & Χ\left(6\right) \end{array}\right]}^{T}\\ J^{-1}\left(\ddot{\chi }-\dot{J}\dot{\theta }\right)\\ v\\ \mu \left(1-u^{2}\right)v-\omega_{0}^{2}u-\alpha u^{3}-\gamma u^{5}+F_{1}cos\omega_{1}t+F_{2}cos\omega_{2}+0.1\left(\left(Χ\left(4\right)+Χ\left(5\right)+Χ\left(6\right)\right)sin\left(Χ\left(1\right)+Χ\left(2\right)+Χ\left(3\right)\right)\right) \end{array}\right]$$

where:

$$\chi ={\left[\begin{array}{ccc} x & y & \theta_{g} \end{array}\right]}^{T};\;\;\;u=x_{D};\;\;v={\dot{x}}_{D}=\frac{\left(Χ\left(1\right)+Χ\left(2\right)+Χ\left(3\right)\right)}{d}$$

$${\ddot{\theta }}_{g}=k\left[2\left(\mu \left(1-u^{2}\right)v-\omega_{0}^{2}u-\alpha u^{3}-\gamma u^{5}+F_{1}cos\omega_{1}t+F_{2}cos\omega_{2}\right)+0.1\left({\dot{\theta }}_{g}sin\theta_{g}\right)\right]$$

## 4. Synchronization of Planar Human Arm System with PD-Scheme 

The second model, which was studied to investigate the chaotic behavior of the planar human arm motion, is the synchronization-based approach. Recently, the control system method was added to the research on the synchronization of the dynamical system [26,27,28]. In this paper, the PD-force control scheme adapted from the control system theory was applied. The synchronization phenomenon in planar repetitive arm motion was obtained by modeling the orientation angle as a biological oscillator using the DVP system employing the PD scheme. The planar human arm system is driven by the chaotic extended DVP system, representing the uncertainty condition during the repetitive movement. 

### Modeling the θ_g_ Trajectories as the DVP Oscillator

Modeling the orientation angle as the single-well DVP oscillator can be obtained as follows:

(31) $${\ddot{\theta }}_{g}=\mu_{s}\left(1-\theta_{g}{}^{2}\right){\dot{\theta }}_{g}-\omega_{0s}^{2}\theta_{g}-\alpha_{s}\theta_{g}{}^{3}+U\left(t\right)$$

where *U(t)* is an external force and $\mu_{s}$, $\omega_{0s}$, $\alpha_{s}$ are constant parameters.

Equation (31) has been used in physics, engineering, biology, and many other subjects and is one of the most studied systems in nonlinear dynamics and chaos [25]. Using the PD scheme, the external force was used as the control input. 

Since the human arm has joint limitations, to keep the trajectories inside the $\theta_{g}$ boundary, the drive system was obtained through the following scheme:

(32) $$x_{m}=\kappa +hx_{D};\;{\dot{x}}_{m}=h{\dot{x}}_{D};{\ddot{x}}_{m}=h{\ddot{x}}_{D}$$

(33) $${\ddot{x}}_{D}=\mu \left(1-x_{D}^{2}\right){\dot{x}}_{D}-\omega_{0}^{2}x_{D}-\alpha_{1}x_{D}^{3}-\gamma x_{D}^{5}+F_{1}cos\omega_{1}t+F_{2}cos\omega_{2}t$$

where κ, *h*, $x_{m}$, and $x_{D}$ are a constant, a scale factor, the drive trajectories, and the extended DVP displacement trajectories, respectively.

By this scheme, the drive system is determined from the chaotic system after mapping through Equation (32). This step was necessary to maintain the drive system, which was obtained from the extended DVP system, lying within the orientation angle boundary. 

Model-based control law of the PD controller can be expressed as follows:

(34) $$M=-K_{p}e_{1}-K_{v}e_{2}\;e_{1}=x_{m}-\theta_{g}\;\;\;e_{2}={\dot{x}}_{m}-{\dot{\theta }}_{g}\;U\left(t\right)=U_{ref}=\left({\ddot{x}}_{m}-M\right)-\mu_{s}\left(1-\theta_{g}^{2}\right){\dot{\theta }}_{g}+\omega_{0s}^{2}\theta_{g}+\alpha_{s}\theta_{g}^{3\;}\;\;={\ddot{x}}_{m}+K_{p}e_{1}+K_{v}e_{2}-\mu_{s}\left(1-\theta_{g}^{2}\right){\dot{\theta }}_{g}+\omega_{0s}^{2}\theta_{g}+\alpha_{s}\theta_{g}^{3}$$

where *M*, *K_v_*, *K_p_*, $e_{1}$, and $e_{2}$ are the controller output, the derivative gain, the proportional gain, the position error, and the velocity error, respectively.

Using the PD scheme, the orientation angle of the open kinematic chain of the human arm was modeled as the DVP oscillator, which could synchronize with other chaotic systems. 

## 5. Results and Discussions

For the numerical experiments, a Lissajous curve was employed as follows:

(35) $$x_{e}\left(\varphi \right)=x_{c}+Asin\left(a\varphi +\delta \right);\;y_{e}\left(\varphi \right)=y_{c}+Bsin\left(b\varphi \right)$$

where *A* and *B* are constant numbers, *a* and *b* are integer values, δ is a positive real number, integer value, and (*x_c_, y_c_*) is the curve center position in the Cartesian coordinate. 

**Figure 4:**
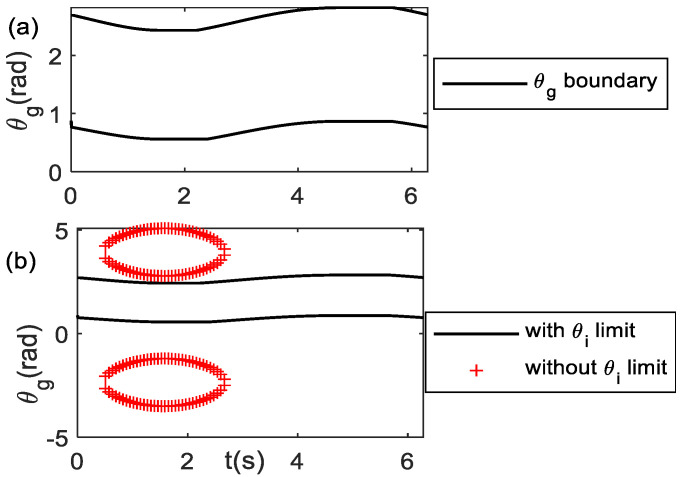
$\theta_{g}$ boundary for one complete cycle *t* = [0, *T_~_*] (**a**) rad (**b**) comparison of $\theta_{g}$ boundary with and without considering *θ_i_* limit.

The end-effector motion of Equation (35) is repeated every *φ =* 2*π* rad so that it has a curve frequency of *Ω = 2π/T_~_* (see Equation (2))*,* with *T_~_* as the period of end-effector motion. Table 1 tabulates the joint limits of the planar human arm model [36]. Using the curve parameter values: *A* = 7, *B* = 7, *a =* 1, *b =* 1, *δ* = 0, and (*x_c_, y_c_*) = (32, 32), the geometry of the end-effector path is a linear curve. Solving Equations (20) and (21) iteratively for (*x_e_, y_e_*) trajectories, Figure 4a shows the $\theta_{g}$ boundary for one cycle of motion for this end-effector path. For the *n*-cycle of motion, the $\theta_{g}$ boundary repeats *n*-time. During the motion to perform the repetitive linear curve, the orientation angle trajectories should lie on its boundary. The area of the $\theta_{g}$ boundary with joint limits reduces as compared to the $\theta_{g}$ boundary without considering the joint limit, as shown in Figure 4b. 

This paper investigates the dynamical behavior of the coupled system when the angular frequency of the end-effector path is the same as the first angular frequency of the extended DVP system: $\Omega =\omega_{1}$. During the repetitive movement, the end-effector path is constrained or fixed so that the initial conditions of $\theta_{i}$ and ${\dot{\theta }}_{i}$ are also constrained. The value of the $\theta_{i}$ and ${\dot{\theta }}_{i}$ initials should be computed from the IK solution. Thus, for all of the discussions in this paper, the initial conditions of the planar arm system are in the form of the initial orientation angle, $\theta_{gi}$ and the initial orientation angle velocity ${\dot{\theta }}_{gi}$. The initial velocities ${\dot{\theta }}_{i}$ are then computed from the first order kinematic differential: ${\dot{\theta }}_{i}=J^{-1}{\dot{\chi }}_{i}$. For scheme-1, this paper uses the initial conditions *(*$\theta_{gi}$, ${\dot{\theta }}_{gi}$) = (1.75, 0) and ($x_{D0}$, ${\dot{x}}_{D0}$) = (0.6, 0.6) for the planar arm system and the DVP system, respectively. The Poincare sections are computed at period points $t=\frac{2\pi }{\Omega }+T_{\sim }$. The Poincare section is computed using 1000 cycles of the repetitive movements, with the first 30% of motions being ignored since they are considered to be a transient response. The heuristic approach proposed by Wiebe and Virgin [37], which works by counting the number of peaks in the Discrete Fourier Transform (DFT), is applied to strengthen the observation. For this heuristic approach, ${\dot{\theta }}_{3}\;$is used as the investigated state using 300 cyclic motions and computed for the last 40% of motions. 

### 5.1. Scheme-1 of Coupled System Model

**Figure 5:**
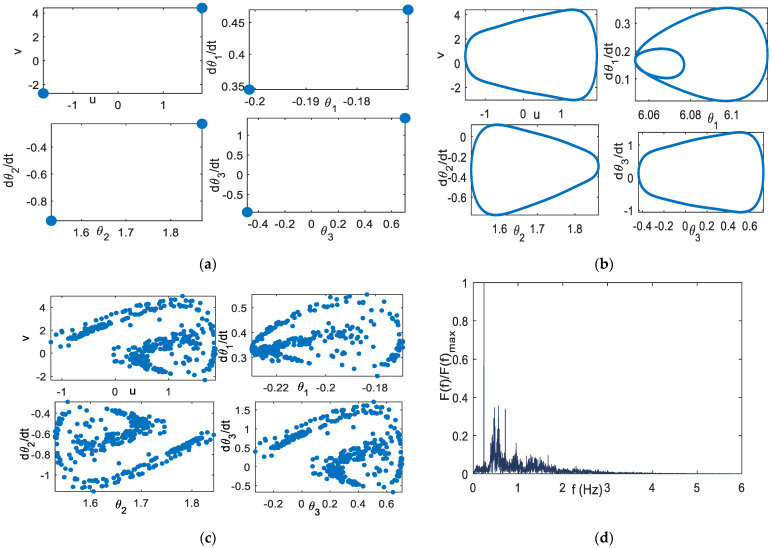
*α* = −3, *F*_1_ = 2, γ = 2, *d* = 0.25 (**a**) Poincare section, period−2: *μ* = 0.1, $\omega_{0}$ = 0.2, *F*_2_ = 3, *ω_1_* = 1, *ω_2_* = 2 (**b**) Poincare section, quasi-periodic: *μ* = 0.1, $\omega_{0}$ = 1, *F*_2_ = 0.1, $\omega_{1}$ = 0.5, $\omega_{2}$ = √5 (**c**) Poincare section, chaotic: *μ* = 1, *ω_0_* = 0.2, *F*_2_ = 0.1, $\omega_{1}$ = 1, $\omega_{2}$ = √5 (**d**) heuristic approach of (**c**).

Without coupling with the planar arm system, the extended DVP system with parameters: *μ* = 0.1, $\omega_{0}$ = 0.2, *α* = −3, *F_1_* = 2, *F_2_* = 3, γ = 2, $\omega_{1}$ = 1, $\omega_{2}$ = 2, with coupling parameter *d* = 0.25, exhibits the period-2 solution. Using these parameter values, the Poincare section of the coupled system is period-2, as shown in Figure 5a. Figure 5b shows the Poincare section of the coupled system using the parameter values: *μ* = 0.1, $\omega_{0}$ = 1, *α* = −3, *F*_1_ = 2, *F*_2_ = 0.1, γ = 2, $\omega_{1}$ = 0.5, $\omega_{2}$ = √5, using *d* = 0.25. It indicates the quasi-periodic solution, the same as the orbit solution without coupling with the planar arm system. Using the parameters: *μ* = 1, $\omega_{0}$ = 0.2, *α* = −3, *F*_1_ = 2, *F*_2_ = 0.1, γ = 2, $\omega_{1}$ = 1, $\omega_{2}$ = √5, and *d* = 0.25, the Poincare section of the coupled system indicates the chaotic orbit, as shown in Figure 5c. The chaotic behavior can be further observed using the heuristic approach proposed by Wiebe and Virgin [37], where there are many number peaks in the DFT, as shown in Figure 5d.

### 5.2. Scheme-2 of Coupled System Model

Scheme-1 has shown that by sharing the velocity value of the planar arm system with the extended DVP oscillator, chaotic behavior is observed when the planar arm system is coupled with the chaotic extended DVP oscillator. However, only the effect of scaling is detected while the dynamical behavior of the coupled system remains the same as the original, extended DVP oscillator. The route to chaos cannot be observed in scheme-1. 

#### k Range Which Exhibits the Chaotic Behavior

**Figure 6:**
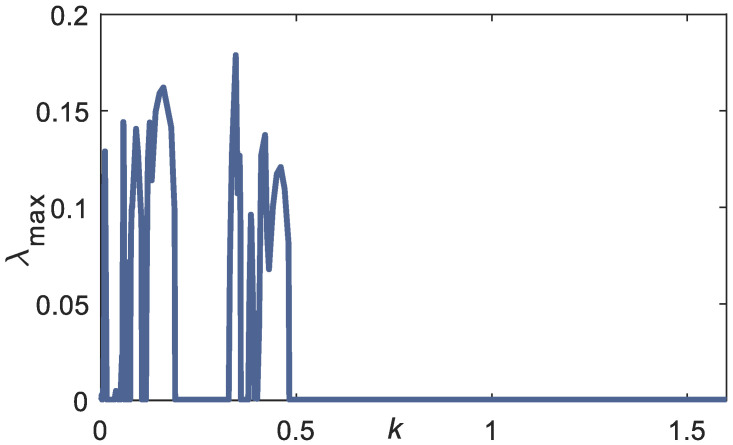
LLE versus coupling constant *k* to track rhythmic linear curve. Fixed parameter: *d* = 0.25, *μ* = 1, $\omega_{0}$ = 0.2, *α* = −3, *F*_1_ = 2, *F*_2_ = 0.1, $\omega_{1}$ =1, $\omega_{2}$ = √5, *l* = [31.5 28.7 10.5] cm. Initial conditions: *(*$\theta_{gi}$,${\dot{\theta }}_{gi}$) = (1.75, 0), ($x_{D0}$, ${\dot{x}}_{D0}$) = (0.6,0.6).

For scheme-2, the chaotic extended DVP system with parameters [24]: *μ* = 1, $\omega_{0}$ = 0.2, *α* = −3, *F*_1_ = 2, *F*_2_ = 0.1, γ = 2, $\omega_{1}$ = 1, $\omega_{2}$ = √5, is employed in the numerical experiment to be coupled with the planar arm system. Without coupling with the planar arm, these parameter values exhibit chaotic behavior in the extended DVP system [24]. Consider fixing the value of *d* = 0.25 and the initial conditions to *(*$\theta_{gi}$, ${\dot{\theta }}_{gi}$)=(1.75, 0) and ($x_{D0}$, ${\dot{x}}_{D0}$)= (0.6,0.6), the *k* range, which yields the chaotic solution, is searched with the searching area 0 ≤ *k* ≤ 2.5. Figure 6 shows the maximum LE of the coupled system with variation in the coupling constant *k*. The LE is computed using Wolf’s algorithm [38] using 20,000 iteration numbers. The variation in coupling constant *k* is observed because it represents the coupling strength between the planar arm system with the environment uncertainty. The effect of coupling strength to the route to chaos is investigated. 

The computation of Jacobian in Wolf’s algorithm is computed using the MATLAB symbolic computation. The maximum positive LE is observed at the weak coupling constant *k, k* < 0.5. The orbit of solutions can be further confirmed using the Poincare map and heuristic approach proposed by Wiebe and Virgin [37]. This heuristic approach works by counting the number of peaks in the Discrete Fourier Transform (DFT). The Poincare sections are computed at period point: $t=\frac{2\pi }{\Omega }+T_{\sim }$. 

**Figure 7:**
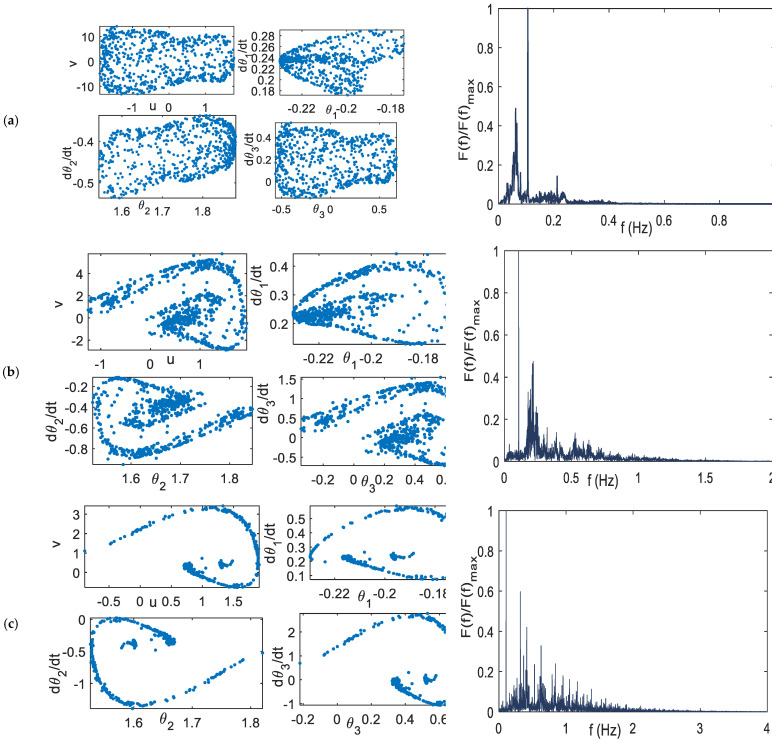
Chaotic orbit, right: Poincare section; left: heuristic approach (**a**) *k* = 0.009 (**b**) *k* = 0.1 (**c**). *k* = 0.35.

From Figure 6, parameter values: *k* = 0.009, *k* = 0.1, and *k* = 0.35, have a positive Largest LE (LLE), which should exhibit the chaotic behavior. The chaotic attractor of these coupling constants can be observed on the left side of Figure 7. The chaotic behavior can be further confirmed using the heuristic approach [37], where there are many numbers of peaks in the DFT result, as shown in the right panel of Figure 7. 

**Figure 8:**
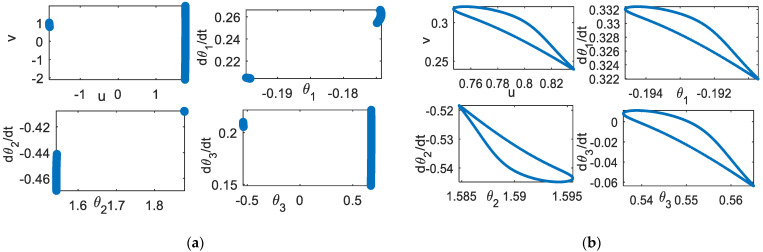
Poincare section (**a**) *k* = 0.007 (**b**) *k* = 0.358.

**Figure 9:**
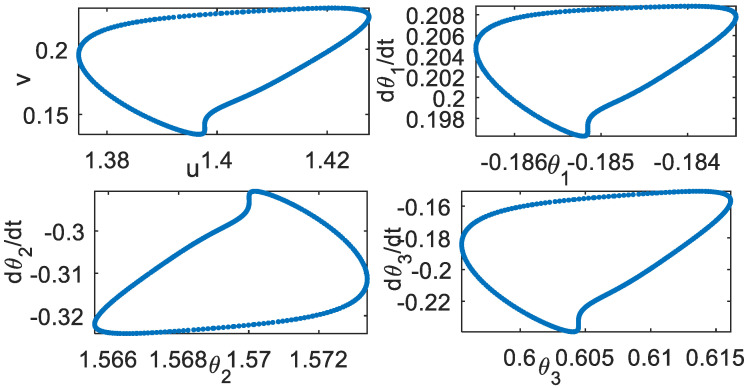
Effect of parameter *d*. *k* = 0.35. Poincare section, *d* = 0.3.

The transformation of the attractor pattern can be observed. For example, using the coupling constant *k* = 0.007 and *k* = 0.358, the orbits are quasi-periodic, as shown in Figure 8. Using the value of *k* = 0.35 and *d* = 0.3, the coupled system exhibits the quasi-periodic solution, as shown in Figure 9. Compared with Figure 7c, changing the value of *d* can yield significantly different orbit solutions.

### 5.3. Sensitivity to Initial Conditions

**Figure 10:**
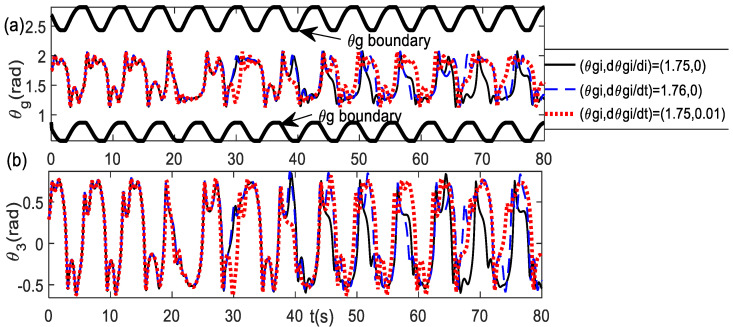
Sensitivity to initial condition with difference value 0.01 only (**a**) $\theta_{g}$ trajectories (**b**) $\theta_{3}$ trajectories.

The chaotic system always exhibits sensitivity to the initial condition. Figure 10 illustrates the trajectory results using scheme-2 with the parameters *d* = 0.25 and *k* = 0.35 for the initial conditions, which have a difference value of 0.01 only. It shows that the trajectories can be significantly different despite the very small difference in the initial conditions. 

### 5.4. Phase Space

**Figure 11:**
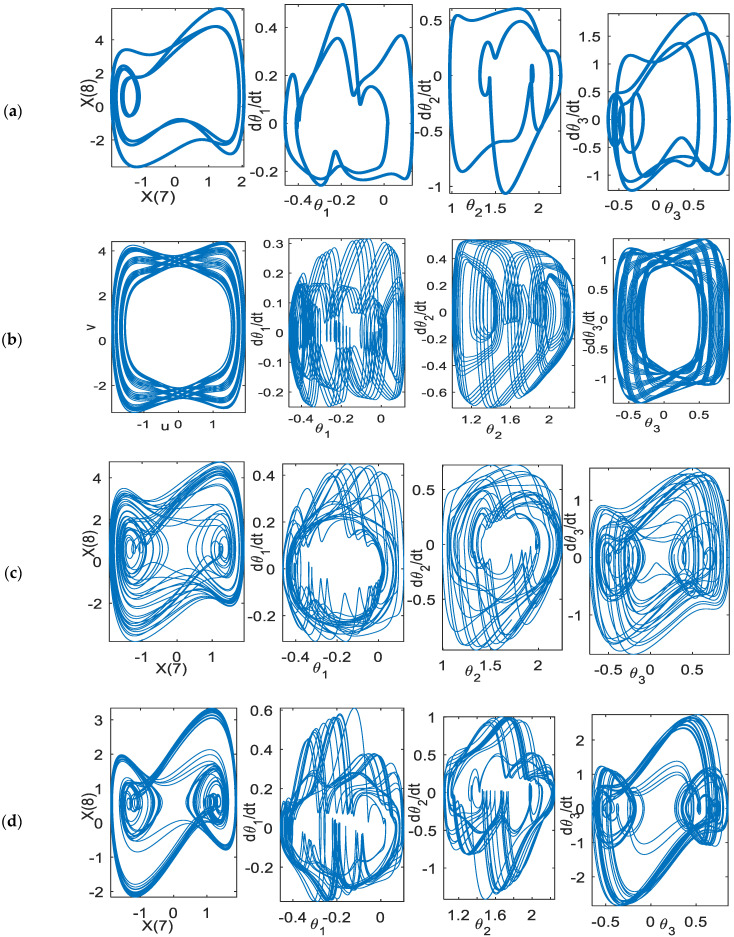
Phase space: *d* = 0.25, *(*$\theta_{gi}$, ${\dot{\theta }}_{gi}$) = (1.75, 0) (**a**) period-2, scheme−1 (**b**) quasi−periodic, scheme-1, (**c**) chaotic, scheme−1 (**d**) chaotic, scheme−2, *k* = 0.35.

Figure 11 shows the phase space of the planar rhythmic arm movement for the periodic, quasi-periodic, and chaotic solutions to track the linear curve. The motion flow differences among these types of solutions can be clearly observed. The period-*n* has only an *n*-flow of motion. Quasi-periodic solutions have a regular flow pattern as compared to chaotic flow, which has a messier geometry. 

### 5.5. System Response of Synchronization Model

The system response of the synchronization model is observed when Equation (32) has the parameter values: *d* = 0.25 and κ = 1.6, as follows:

$$x_{m}=1.6+0.25x_{D};\;{\dot{x}}_{m}=0.25{\dot{x}}_{D};\;{\ddot{x}}_{m}=0.25{\ddot{x}}_{D}$$

**Figure 12:**
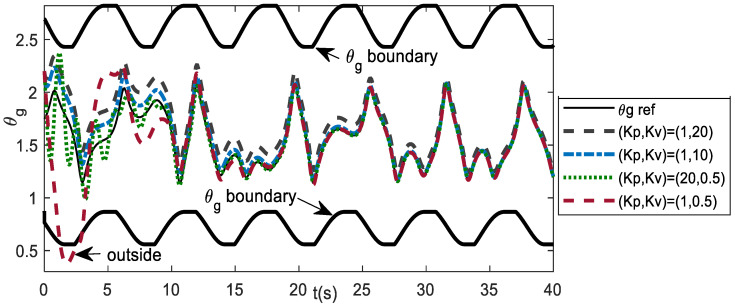
Effect of gains *K_p_*, *K_v_* for initial condition *(*$\theta_{gi}$, ${\dot{\theta }}_{gi}$) = (2.2, −2), *(*$x_{D0}$, ${\dot{x}}_{D0}$) = (0.6,0.6). The gains should yield the $\theta_{g}$ trajectories inside its boundary.

The parameter values of the extended DVP that was used as the drive system are: *μ* = 1, $\omega_{0}$ = 0.2, *α* = -3, *F*_1_ = 2, *F*_2_ = 0.1, γ = 2, $\omega_{1}$ = 1, $\omega_{2}$ = √5, the same as scheme-2 of the coupled system model. For numerical experiments, the parameters of the response systems are *μ_s_* = 0.2, *ω_0s_* = 0, and *α_s_* = 1. As in the coupled system model, principally, the trajectories are feasible if the $\theta_{g}$ trajectories are inside the $\theta_{g}$ boundary because of the joint limits. The initial conditions and gain parameters should be chosen in such a way that the trajectories lie within the $\theta_{g}$ boundary. The trajectory response depends on the values of the initial conditions and gain parameters *K_p_* and *K_v_*. Figure 12 shows the effect of the *K_p_* and *K_v_* values on the system response for different initial conditions of $\theta_{gi}$. The value of the gains, which have the $\theta_{g}$ trajectories outside of the boundary, is unfeasible since it contains the joint angles that are beyond the operational area. For example, *K_p_* = 1 and *K_v_* = 0.5 yield unfeasible $\theta_{g}$ trajectories.

**Figure 13:**
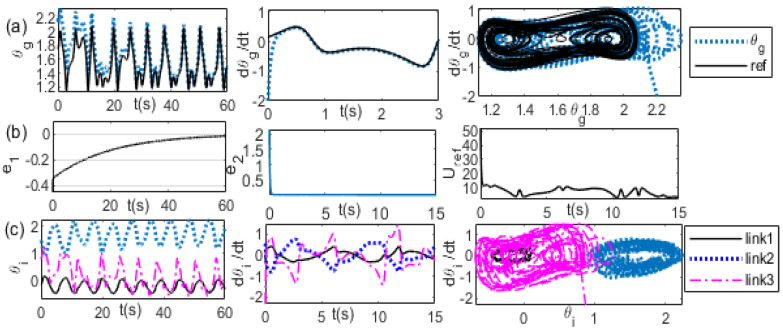
($\theta_{gi}$, ${\dot{\theta }}_{gi}$) = (2.2, −2), *(*$x_{D0}$, ${\dot{x}}_{D0}$) = (0.6,0.6), *K_p_* = 1, *K_v_* = 20 (**a**) left: $\theta_{g}$, middle: ${\dot{\theta }}_{g}$, right: $\theta_{g}-{\dot{\theta }}_{g}$ (**b**) left: $\theta_{g}$ error, middle: ${\dot{\theta }}_{g}$ error, right: *U_ref_* (**c**) left: $\theta_{i}$, middle: ${\dot{\theta }}_{i}$, right: $\theta_{i}-{\dot{\theta }}_{i}$ trajectories.

Figure 13 illustrates the system response of the synchronization model for (*K_p_*, *K_v_)* = (1, 20). A comparison of the reference of the $\theta_{g}$ trajectories with the actual $\theta_{g}$ trajectories, the reference of the $\theta_{g}$ velocity with the actual $\theta_{g}$ velocity, and $\theta_{g}-{\dot{\theta }}_{g}$ between the reference and actual trajectories are shown sequentially in Figure 13a from the first panel to the third panel. The trajectories of the position error, the velocity error, and the external force are shown sequentially in Figure 13b from the first panel to the third panel. A comparison of the joint angle trajectories of the first, second, and third links is shown in the first panel of Figure 13c. A comparison of the velocity trajectories of the first, second, and third links is shown in the second panel of Figure 13c. A comparison of $\theta_{i}-{\dot{\theta }}_{i}$ of the first link, second link, and the third link is shown in the third panel of Figure 13c.

**Figure 14:**
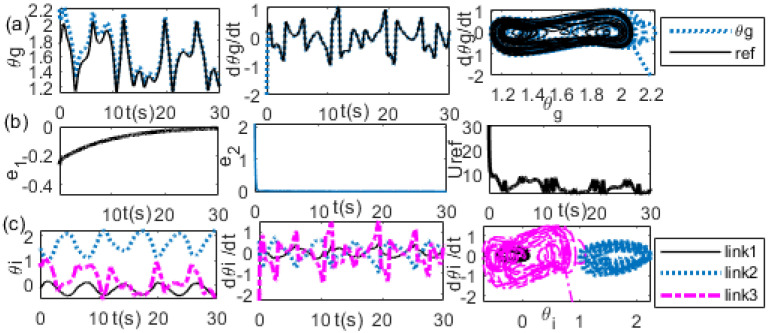
($\theta_{gi}$, ${\dot{\theta }}_{gi}$) = (2.2, −2), *(*$x_{D0}$, ${\dot{x}}_{D0}$) = (0.6,0.6), *K_p_* = 1, *K_v_* = 10 (**a**) left: $\theta_{g}$, middle: ${\dot{\theta }}_{g}$, right: $\theta_{g}-{\dot{\theta }}_{gi}$ (**b**) left: $\theta_{g}$ error, middle: ${\dot{\theta }}_{g}$ error, right: *U_ref_* (**c**) left: $\theta_{i}$, middle: ${\dot{\theta }}_{i}$, right: $\theta_{i}-{\dot{\theta }}_{i}$ trajectories.

**Figure 15:**
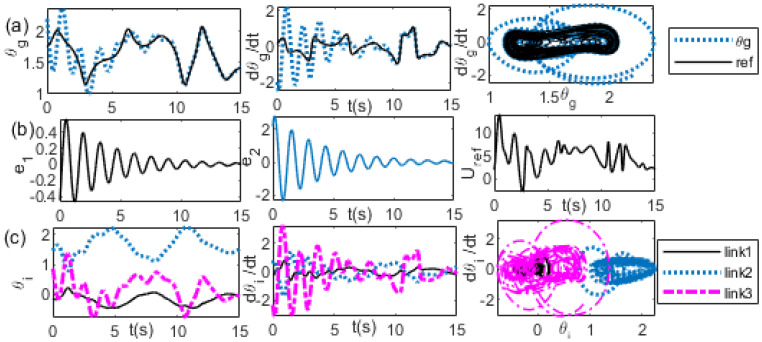
($\theta_{gi}$, ${\dot{\theta }}_{gi}$ ) = (2.2, −2), *(*$x_{D0}$, ${\dot{x}}_{D0}$ ) = (0.6,0.6), *K_p_* = 1, *K_v_* = 10 (**a**) left: $\theta_{g}$, middle: ${\dot{\theta }}_{g}$, right: $\theta_{g}-{\dot{\theta }}_{gi}$ (**b**) left: $\theta_{g}$ error, middle: ${\dot{\theta }}_{g}$ error, right: *U_ref_* (**c**) left: $\theta_{i}$, middle: ${\dot{\theta }}_{i}$, right: $\theta_{i}-{\dot{\theta }}_{i}$ trajectories.

It shows that it needs a longer time of transient response before the synchronization is achieved. By reducing the value of *K_v_* to *K_v_* = 10, the transient time becomes faster than that of the previous value, as shown in Figure 14. A comparison of the reference of the $\theta_{g}$ trajectories with the actual $\theta_{g}$ trajectories, the reference of the $\theta_{g}$ velocity with the actual $\theta_{g}$ velocity, and $\theta_{g}-{\dot{\theta }}_{g}$ between the reference and the actual trajectories are shown sequentially in Figure 14a from the first panel to the third panel. The trajectories of the position error, the velocity error, and the external force are shown sequentially in Figure 14b from the first panel to the third panel. A comparison of the joint angle trajectories of the first, second, and third links is shown in the first panel of Figure 13c. A comparison of the velocity trajectories of the first, second, and third links is shown in the second panel of Figure 13c. A comparison of $\theta_{i}-{\dot{\theta }}_{i}$ of the first link, second link, and the third link is shown in the third panel of Figure 14c. However, further reducing the value of *K_v_* to 0.5, the transient responses are outside the boundary, as has been observed in Figure 12. Increasing the value of *K_p_* to 20, e.g., (*K_p_*, *K_v_)* = (20, 0.5), the transient response shows more oscillation than the previous value, as shown in Figure 15. A comparison of the reference of the $\theta_{g}$ trajectories with the actual $\theta_{g}$ trajectories, the reference of the $\theta_{g}$ velocity with the actual $\theta_{g}$ velocity, and $\theta_{g}-{\dot{\theta }}_{g}$ between the reference and the actual trajectories are shown sequentially in Figure 15a from the first panel to the third panel. The trajectories of the position error, the velocity error, and the external force are shown sequentially in Figure 15b from the first panel to the third panel. A comparison of the joint angle trajectories of the first, second, and third links is shown in the first panel of Figure 15c. A comparison of the velocity trajectories of the first, second, and third links is shown in the second panel of Figure 15c. A comparison of $\theta_{i}-{\dot{\theta }}_{i}$ of the first link, second link, and the third link is shown in the third panel of Figure 15c.

**Figure 16:**
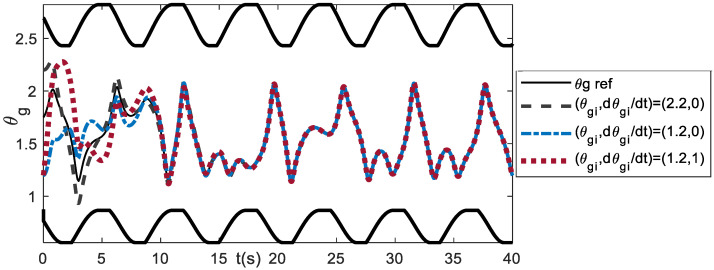
Effect of initial conditions ($\theta_{gi}$, ${\dot{\theta }}_{gi}$ ) for *K_p_* = 1 and *K_v_* = 0.5.

In addition to the gain parameters, the initial conditions of the orientation angle $\theta_{g}$, and velocities ${\dot{\theta }}_{gi}$, should be chosen in such away so that the generated $\theta_{g}$ trajectories lie within the $\theta_{g}$ boundary. It has been shown that by using *K_p_* = 1 and *K_v_* = 0.5, with *(*$\theta_{gi}$, ${\dot{\theta }}_{gi}$) = (2.2, −2), the $\theta_{g}$ trajectories are outside the boundary. Changing the initial conditions of velocities ${\dot{\theta }}_{gi}$ to 0, e.g., *(*$\theta_{g}$, ${\dot{\theta }}_{gi}$) = (2.2, 0), the transient responses are inside the $\theta_{g}$ boundary, as shown in Figure 16. Other initial conditions that can be chosen for (*K_p_*, *K_v_)* = (1, 0.5), are *(*$\theta_{g}$, ${\dot{\theta }}_{gi}$) = (1.2, 0) and *(*$\theta_{g}$, ${\dot{\theta }}_{gi}$) = (1.2, 1). 

The results in this section have shown that synchronization with the chaotic systems is possible through the PD scheme when the orientation angle is driven by the chaotic, extended DVP system; however, as in the coupled system model, it should be noted that the system response should lie inside the $\theta_{g}$ boundary since the human arm has joint limitations. This goal can be achieved by adjusting the gain parameters and the initial conditions so that the system response has orientation angle trajectories inside the $\theta_{g}$ boundary. 

### 5.6. Discussions

**Figure 17:**
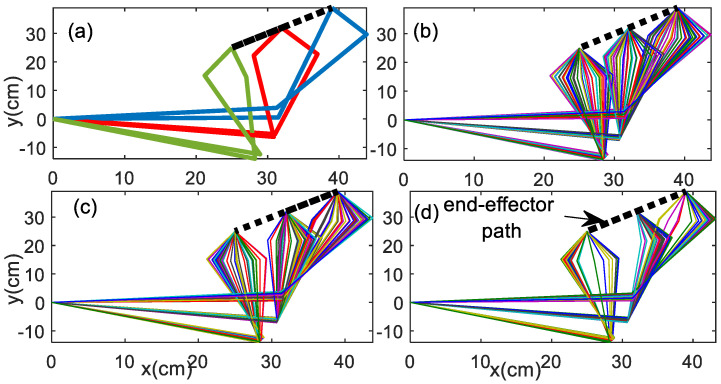
Posture 300 cyclic motions, plotted for last 10% of linear path motion (**a**) period−2, scheme−1 (**b**) quasi−periodic, scheme−1 (**c**) chaotic scheme−1 (**d**) chaotic scheme−2.

Movement variability can be further observed using the link configurations. The posture can be computed from the joint angle trajectories obtained from the system response. Figure 17 illustrates the posture of the rhythmic planar arm movement to track the linear curve for periodic, quasi-periodic, and chaotic behaviors. The postures are computed at 300 cyclic motions and plotted for the last 10% of motions. Period-*n* reveals the *n*-possible posture at one instantaneous end-effector point. The quasi-periodic has a little more posture variability as compared with the periodic solution, but it is still less posture variability as compared with the chaotic solutions. Keeping the same angle cycle to cycle during the rhythmic motion is potentially an uncomfortable action for the muscles of the arm.

The research question was whether chaotic behavior is indeed possible to present in repetitive human arm motions. The present study has shown that the coupling scheme and synchronization can be used to model the mechanism by which chaotic trajectories appear in the repetitive motions of the human arm in the planar plane. The chaotic nonlinear oscillator has been used to represent the uncertainty conditions that interact with the planar arm system during rhythmic arm movements. Depending on the value of the coupling parameter and the parameter of the nonlinear oscillator, the system response of the coupled model shows very interesting dynamical behavior, where different types of solutions from the periodic, the quasi-periodic, to the chaotic can be observed. The effect of the coupling scheme is more remarkable in scheme-2, where the *k* range, which exhibits chaotic behavior, can be observed. The coupled system model has shown that the advanced bidirectional coupling scheme is necessary for exhibiting the route to chaos. The advanced coupling scheme is obtained by adding the parameter *k*, as Equation (31),in addition to parameter *d*. The chaotic solution is observed at the very small value of parameter *k*. 

By the PD-scheme model, the chaotic behavior of the planar repetitive arm movement presents when the planar arm system is driven by chaotic trajectories. The system response follows the drive system with the transient response depending on the values of the gains *K_p_* and *K_v_*. The PD gain parameters are used to maintain the joint angle trajectories under the joint angle operational area by keeping the trajectories of the orientation angle inside its boundary during the transient response. The value of initial conditions, $\theta_{gi}$ and ${\dot{\theta }}_{gi}$, should be adjusted or chosen in such a way that the result of the $\theta_{g}$ trajectories are inside the $\theta_{g}$ boundary. 

Prior research on rhythmic human arm movements using the nonlinear oscillator model did not explicitly employ the model of the human body system. The approaches developed in this paper, which are the coupling scheme model and the synchronization between the planar human arm system and the chaotic system representing the uncertainty condition during the rhythmic movement, employ the kinematic differential equation of the human arm system. Thus, it will provide a better understanding of the movement variability of the open kinematic chain of the human body system instead of using only the nonlinear oscillator without considering the human body system. 

Two important remarks should be addressed to be successful in the numerical computation to solve the developed ODE system. Firstly, the initial condition of $\theta_{i}$ and ${\dot{\theta }}_{i}$ should be computed from the orientation angles $\theta_{gi}$ and ${\dot{\theta }}_{gi}$ because of the end-effector hand constraint. These initial conditions cannot be chosen arbitrarily as in the common ODE system because the end-effector path has been fixed. Secondly, the system response should lie on the orientation angle boundary due to the joint angle limits of the human arm. Failure to perform the first point means that the ODE solver will face computational failure, and the trajectories will become unfeasible for tracking the end-effector path if the orientation angle trajectories are outside the boundary, although the ODE solver seems successful in the computation. 

Using the nonlinear dynamics approach, which involves the nonlinear oscillator, to model the human biological system, the details of the parameter values of the ODE system are different from person to person depending on the person’s health. The values can be obtained through experimental investigation and the estimation of the biomechanics data. Mathematically, the developed approach explores movement variability in the form of the orientation angle variable. 

The results of the coupled system model have clearly shown that the chaotic solutions are possible to present when the end-effector hand performs the periodic motion, i.e., the Lissajous path. The ODE solutions, whether they are periodic, quasi-periodic, or chaotic, depend on the parameter value of the coupling constant. It shows that the chaotic structures have been observed at the small coupling constant, i.e., *k* < 0.5. The coupling constant represents the strength of the coupling between the planar arm system and the environment uncertainty. This result possibly supports the Goldberg hypotheses, stating that chaotic behavior represents the healthy state of the human body [11]. The weak coupling strength can be considered as the healthy condition of the human musculoskeletal system that is resilient to the environmental uncertainty associated with daily stressors. The negative emotions that may come at anytime in daily life, such as sadness, sorrow, fear, jealousy, and anger, will not have too much of an effect on the performance of the human musculoskeletal system. 

For the synchronization model, the orientation angle is modeled as the nonlinear oscillator, which can synchronize with other dynamical systems. The results of the synchronization model can be potentially beneficial to the study of the phenomenon related to pathological rhythm, such as Parkinson’s disease, where patients have lost the ability to control body movements. It is known that exceeding synchronization can lead to pathological rhythms [39,40,41,42,43]. Further experimental study on the biomechanics of the repetitive end-effector hand movement is necessary to support these conclusions and to further explore the developed approach for analyzing the issue of a healthy movement system. The experimental phase is also part of the authors’ forthcoming research. 

## 6. Conclusions

The results showed that chaotic behavior was possible to present when the planar arm system was coupled with the chaotic system, i.e., the extended DVP oscillator, through a certain scheme. The ODE system of the planar arm was established from the robotic motion approach, and the nonlinear oscillator was employed to represent the uncertainty condition during the rhythmic movement. Using the ODE-based model, dynamical behavior can be clearly observed using nonlinear tools from chaos theory. An advanced coupling scheme was necessary to exhibit the route to chaos, i.e., the scheme-2 coupled system model. By varying the coupling constant *k*, the chaotic behavior has been observed at the weak coupling constant. The synchronization phenomenon between the planar arm system and the nonlinear oscillator has also been studied using the PD-scheme method. The results show that the synchronization of the planar arm system with the chaotic system was possible via the PD scheme when the orientation angle was driven by the chaotic, nonlinear oscillator; however, as in the coupled system model, it should be noted that the system response should lie inside the $\theta_{g}$ boundary since the human arm has joint limitations. This goal can be achieved by adjusting the gain parameters and the initial conditions in such a way that the system response has the orientation angle trajectories inside the $\theta_{g}$ boundary. Movement variability was present in the planar arm system in the form of the orientation angle variable, and by exploring this variable, chaotic behavior can be observed. 

## Figures and Tables

**Table 1:** Parameter of the planar arm system [36].

*l_1_* (Upper Arm)	*l_2_* (Forearm)	*l_3_* (Hand)	*θ_i_* Limits
31.5 cm	28.7 cm	10.5 cm	$\theta_{1}$ = [ −140°, 90°]; $\theta_{2}$ = [0°, 145°]; $\theta_{3}$ = [ −70°, 90°]

## Data Availability

Not applicable.

## Nomenclature

A	Constant number
*A_p_*	Amplitude of $c_{2}$
a	Integer value
B	Constant number
b	Integer value
$c_{2}$	Cosine of second joint angle
*d*	Coupling parameter
$D_{\theta_{i}}$	Domain or the operational area of the *i*th joint angle
*e_i_*	Error
*e_1_*	Position error
*e_2_*	Velocity error
*F_i_*	Real parameter of extended DVP oscillator
f(φ)	Parametric function
g(φ)	Parametric function
*h*	Scale factor in synchronization approach
*J*	Jacobian
*k*	Coupling parameter
*K_p_*	Proportional gain
*k_p_*	Vertical shift of $c_{2}$
*K_v_*	Derivative gain
*l_i_*	*i*th length of the open kinematic chain
*M*	Controller output
R	Real number
*r*	Radius
$s_{2}$	Sine of second joint angle
*t*	Time
*T_~_*	Period time
*U(t)*	External force of single well DVP oscillator
Χ	State variable of coupled system
$x_{D}$	Displacement of extended DVP oscillator
$x_{D0}$	Initial position of extended DVP oscillator
${\dot{x}}_{D0}$	Initial velocity of extended DVP oscillator
$x_{m}$	Drive trajectories of synchronization approach
*(x, y)*	Actual end-effector position
*(x_c_, y_c_)*	Curve center
(x_e_, y_e_)	Target end-effector position
*α*	Real parameter of extended DVP oscillator
$\alpha_{s}$	Constant parameter of single well DVP oscillator
*γ*	Real parameter of extended DVP oscillator
δ	Positive real number
$\theta_{g}$	Orientation angle
$\theta_{i}$	Joint angle of *i*th link
$\theta_{imin}$	Minimum joint angles of *i*th link
$\theta_{imax}$	Maximum joint angles of *i*th link
$\theta_{gi}$	Initial orientation angle
κ	Constant value of PD synchronization
${\dot{\theta }}_{i}$	First derivative of $\theta_{i}$
$\dot{\theta_{gi}}$	Initial orientation angle velocity
$\ddot{\theta_{i}}$	Second derivative of $\theta_{i}$
*μ*	Real parameter of extended DVP oscillator
$\mu_{s}$	Constant parameter of single well DVP oscillator
*φ* *_p_*	Phase shift of $c_{2}$
φ	Angle of curve
χ	State variables of inverse kinematics
*Ω*	Frequency of curve
$\omega_{0}$	Real parameter of extended DVP oscillator
$\omega_{0s}$	Constant parameter of single well DVP oscillator
$\omega_{i}$	Real parameter of extended DVP oscillator
$\partial \theta_{g}$	Boundary of orientation angle

## Appendix A

(A1)
$$w_{x}=x-l_{3}cos(\theta_{g});\;w_{y}=y-l_{3}sin(\theta_{g})$$

(A2)
$$c_{2}=\frac{\left(w_{x}{}^{2}+w_{y}{}^{2}-l_{1}{}^{2}-l_{2}{}^{2}\right)}{2l_{1}l_{2}}$$

The first and third joint angles are expressed as follows:

(A3) $$\Delta =w_{x}{}^{2}+w_{y}{}^{2};\;s_{1}=\frac{\left(l_{1}+l_{2}c_{2}\right)w_{y}-\left(l_{2}s_{2}w_{x}\right)}{\Delta };\;s_{1}=\frac{\left(l_{1}+l_{2}c_{2}\right)w_{y}-\left(l_{2}s_{2}w_{x}\right)}{\Delta }$$

(A4) $$\theta_{1}=atan2\left(s_{1},c_{1}\right)$$

(A5) $$\theta_{3}=\theta_{g}-\theta_{3}-\theta_{1}$$

where $\theta_{1}$, $\theta_{3}$*_,_* $c_{1}$, and $s_{1}$ are the first joint angles, third joint angle, cosine of $\theta_{1s}$, and sine of $\theta_{1s}$, respectively.

## Appendix B

From Equation (9), we can define $c_{2}$ as a function of [*A_p_, k_p_, ϕ_p_,* $\theta_{g}$]:

$$c_{2}=f\left(A_{p}\left(x,y\right),k_{p}\left(x,y\right),\phi_{p}\left(x,y\right),\theta_{g}\right)$$

$$\;\;\;=A_{p}\left(x,y\right)cos\left(\theta_{g}-\phi_{p}\left(x,y\right)\right)+k_{p}\left(x,y\right)$$

Derivative of $c_{2}$ with reference to [*A_p_, k_p_, ϕ_p_,* $\theta_{g}$ ] is:

(A6) $$\frac{dc_{2}}{dt}=\frac{\partial c_{2}}{\partial A_{p}}\frac{dA_{p}}{dt}+\frac{\partial c_{2}}{\partial k_{p}}\frac{dk_{p}}{dt}+\frac{\partial c_{2}}{\partial \phi_{p}}\frac{d\phi_{p}}{dt}+\frac{\partial c_{2}}{\partial \theta_{g}}\frac{d\theta_{g}}{dt}$$

Calculate all parts of above equation:

(A7) $$\frac{\partial c_{2}}{\partial A_{p}}=cos\left(\theta_{g}-\phi_{p}\right);\;\frac{\partial c_{2}}{\partial k_{p}}=1\;\frac{\partial c_{2}}{\partial \phi_{p}}=-A_{p}sin\left(\theta_{g}-\phi_{p}\right);\;\frac{\partial c_{2}}{\partial \theta_{g}}=A_{p}sin\left(\theta_{g}-\phi_{p}\right)\;\frac{dA_{p}}{dt}=\frac{\partial A_{p}}{\partial x}\frac{dx}{dt}+\frac{\partial A_{p}}{\partial y}\frac{dy}{dt}=\frac{l_{3}}{l_{1}l_{2}}\frac{x}{\sqrt{x^{2}+y^{2}}}\frac{dx}{dt}+\frac{l_{3}}{l_{1}l_{2}}\frac{x}{\sqrt{x^{2}+y^{2}}}\frac{dy}{dt}\;\frac{dk_{p}}{dt}=\frac{\partial k_{p}}{\partial x}\frac{dx}{dt}+\frac{\partial k_{p}}{\partial y}\frac{dy}{dt}=\frac{x}{l_{1}l_{2}\sqrt{x^{2}+y^{2}}}\frac{dx}{dt}+\frac{y}{l_{1}l_{2}\sqrt{x^{2}+y^{2}}}\frac{dy}{dt}$$

Substitute the above equations into Equation (B1) and Equation (15) and there will be components of *x, y*, and $\theta_{g}$, which are components of partial derivatives of $\theta_{2}$, and Equation (19) can be obtained.
